# Electronic Imaging in Colonoscopy: Clinical Applications and Future Prospects

**DOI:** 10.1007/s11938-016-0075-1

**Published:** 2016-02-29

**Authors:** R. Rameshshanker, Ana Wilson

**Affiliations:** Imperial College London, Wolfson Endoscopy Unit, St Mark’s Hospital, Watford Road, Harrow, HA1 3UJ UK

**Keywords:** Electronic chromoendoscopy, NBI, FICE, i-Scan, Diminutive polyps, Optical diagnosis

## Abstract

Electronic chromoendoscopy (EC) is an equipment-based technology which could be easily activated by push of a button. There are four EC techniques available for use at present: narrow band imaging (NBI), i-Scan, flexible spectral chromoendoscopy and blue laser imaging. Out of the four techniques, NBI has been extensively evaluated for the detection and characterization of dysplasia in colonic polyps and dysplasia associated with inflammatory bowel disease. In this review, we will focus on the new developments and applications of EC.

## Introduction

Colorectal cancer (CRC) is one of the commonest causes of significant morbidity and mortality in the western world. It is the second most common cause of cancer-related mortality in Europe and the fourth most common cause worldwide [1]. Sporadic CRC arises from adenomatous polyps in a well described adenoma-carcinoma sequence [2]. Approximately, 15–30 % of CRC arise via serrated pathway from precursor lesions and serrated adenomas [3]. Familial CRC and CRC associated with inflammatory bowel disease also arise from dysplastic lesions which can be detected at colonoscopy.

Colonoscopy is the most valuable weapon in the fight against CRC. The efficiency of CRC prevention with colonoscopy depends on complete and careful inspection of colonic mucosa for dysplastic lesions and their successful removal before they progress to cancer [4]. Various techniques have been developed to improve the detection and characterization of dysplastic lesions including chromoendoscopy, electronic chromoendoscopy and autofluorescence [5•, 6•].

Electronic chromoendoscopy (EC) refers to endoscopic imaging technologies which provide detailed contrast enhancement of the mucosal surface and blood vessels. They provide an alternative to conventional chromoendoscopy and improve mucosal visualization of surface and vascular structures through the use of optical filters or software-driven post-image processing [6•, 7]. EC technologies include narrow band imaging (NBI) (Olympus Medical Co., Tokyo, Japan), flexible spectral imaging colour enhancement (FICE) (Fujinon, Fujifilm Co., Saitama, Japan), i-Scan (Pentax Endoscopy, Tokyo, Japan) and blue laser imaging (BLI) (Fujinon, Fujifilm Co., Saitama, Japan).

NBI is an endoscopic optical image enhancement technology. It is based on the penetration properties of light, which is directly proportionate to wavelength. Because most of the NBI light is absorbed by the blood vessels in the mucosa, the resulting image emphasizes the blood vessels in sharp contrast with the non-vascular structures in the mucosa. FICE is a digital imaging post-processing technique, in which white light endoscopic images from the video processor are altered to display a composite colour-enhanced image in real time. i-Scan (Penatax) is similarly a software-based digital, post-processing image enhancement technology. It provides enhanced images of the mucosal surface and the blood vessels through post-image processing. i-Scan enhances contrast and thereby mucosal surface detail including enhanced mucosal surface texture and sharpened views of surface vessels, thereby improving the visibility of blood vessels [6•].

NBI is the most extensively evaluated EC technique in patients undergoing screening or surveillance colonoscopy.

In this review, we focus on the latest developments in the field of electronic chromoendoscopy during colonoscopy, applications and future directions.

## Electronic chromoendoscopy and detection of dysplasia

### Adenoma detection

Adenoma detection rate (ADR) is one of the key quality indicators of colonoscopy and has been shown to inversely correlate with post-colonoscopy interval cancers [8].

### NBI

NBI is the most extensively evaluated EC modality in terms of adenoma detection. Multiple randomized controlled trials showed there was no significant difference in adenoma detection of NBI when compared to white light endoscopy (WLE). East and colleagues randomized 214 patients to examination with NBI or WLE [9]. They showed there was no significant difference in ADR between NBI and WLE (OR 1.4, 95 % CI 1.52–4.63, *p* = 0.26). However the detection of flat adenomas were significantly better with NBI (CR 1.92, 95 % CI 1.07–3.44, *p* = 0.003) [9]. Similar results were described in the study performed by Ikematsu et al. [10] in a multicentre, tandem study that randomized 813 patients to undergo either NBI followed by WLE or WLE followed by NBI. They detected no significant difference in ADR (42.3 % for NBI and 42.5 % for WLE).

Meta-analyses comparing WLE and NBI for the detection of adenomas are summarized in Table 1 [11–15]. Overall, the superiority of NBI over WLE for adenoma detection is not universal and not convincing. One meta-analysis showed that NBI performed better in detecting flat adenomas (RR 1.96, 95 % CI 1.09–3.52, *p* = 0.02) [11].

**Table 1:** Meta-analysis comparing NBI and WLE

Author	Year of publication	No. of studies	Total no. of patients	ADR
Jin et al. [11]	2011	08	3049	RR 1.09 (95 % CI 1.00–1.19)
Dinesen et al. [12]	2012	07	2936	RR 1.06 ( 95 % CI 0.92–1.16)
Nagorni et al. [13]	2012	08	3673	RR 0.94 (95 % CI 0.87–1.02)
Pasha et al. [14]	2012	09	3059	OR 1.01 (95 % CI 0.74–1.37)
Omata et al. [15]	2014	14	5074	RR 1.03 (95 % CI 0.96–1.11)

*RR* relative ratio, *OR* odds ratio, *CI* confidence interval

A recent study comparing new-generation NBI (Olympus Exera HQ190) and high-definition (HD) WLE in a randomized trial with tandem colonoscopy showed encouraging results for NBI [16••]. ADR were significantly better in the NBI group when compared to WLE (48.3 and 34.4 %, *p* = 0.01). Similarly, polyp detection rate was better with NBI (61.1 and 48.3 %, *p* = 0.02).

### Sessile serrated lesions

These polyps could be easily missed due to the subtle appearance (pale colour, poorly defined edges and the presence of mucus on the surface) [17••]. Therefore, endoscopists need to spend additional time to carefully examine the mucosa for sessile serrated adenomas/polyps (SSA/P) especially in the right colon.

NBI has the potential to improve the detection of subtle lesions. Recently, Rex and colleagues performed a randomized controlled trial involving 804 patients to determine the impact of new-generation NBI (Olympus 190 series) to detect SSA/P [18••]. Although numerically more SSA/P proximal to the sigmoid colon of any size were detected in NBI group when compared to WLE group (204 and 158, *p* = 0.085), this was not statistically significant.

### FICE

There is limited data available on the use of FICE. Two prospective, randomized, controlled trials that compared FICE with WLE failed to show any improvement in polyp detection or adenoma detection. Aminalai et al. recruited 637 patients into each group [19]. There was no difference in polyp detection (31.9 % with FICE and 27.7 % with WLE, *p* = 0.10) or ADR (28 % each, *p* = 0.95). The second trial performed by Pohl et al. involving 764 patients did not demonstrate an improvement in adenoma detection with FICE compared to WLE (236 and 271, *p* = 0.92) [20]. A randomized, tandem study in average-risk patients undergoing screening colonoscopy compared WLE with either NBI or FICE [21]. Chung et al. recruited 1650 subjects (550 in each group). FICE did not show any increment in ADR when compared with WLE or NBI (25.3 and 24.5 %, *p* = 0.75). Similarly, a meta-analysis conducted by Omata and colleagues, comparing FICE/i-Scan showed no benefit over WLE (RR 1.09, 95 % CI 0.97–1.23) [15].

### i-Scan

The studies using i-Scan in the detection of adenomas showed mixed results. Hoffmann et al. highlighted a superior ADR with i-Scan when compared to WLE (38 and 13 %, *p* < 0.0001). The detection rate for flat adenomas was higher with i-Scan compared to WLE (58 and 23 %, *p* < 0.0001) [22]. However, the very low ADR in the white light group in this study may have exaggerated the potential benefit of i-SCAN. Two further prospective, tandem studies showed conflicting results. Once again, Hoffmann et al. showed significantly low adenoma miss rate with i-Scan compared to WLE (30 and 62.5 %, *p* < 0.001) [23] whereas another back to back study demonstrated no significant difference in ADR (36.5 and 31.9 %, *p* = 0.54) and adenoma miss rate (19.3 and 22.9 %, *p* = 0.69) comparing WLE and i-Scan [24]. The largest prospective, randomized trial comparing i-Scan with HD WLE involving 1936 patients showed promising results [25••]. Polyps and adenomas were consistently detected more frequently in the i-Scan cohort; 56 % of the i-Scan group patients had a polyp of any type detected compared to 47 % in the control group (*p* = 0.03). Adenomas were detected more frequently in the i-Scan group compared to the WLE group (33 and 27 %, *p* = 0.033). Significantly, more diminutive adenomas were found in the i-Scan group compared to the control group (40.2 and 28.0 %, *p* < 0.01) [25••].

### BLI

To date, there is no published data available on the use of BLI to improve adenoma detection.

In summary, EC especially NBI and i-Scan may improve the detection of diminutive polyps. However, none of the modalities demonstrated consistent improvement in ADR, and, therefore, the routine use of EC is not currently recommended for routine screening or surveillance colonoscopy.

### Electronic chromoendoscopy in inflammatory bowel disease

Long-standing history of ulcerative colitis and Crohn’s colitis are well recognized risk factors for the development of CRC. The cancer risk increases with extent, duration and degree of inflammation, presence of primary sclerosing cholangitis and family history of CRC [26–28].

Therefore, regular surveillance in IBD population is recommended by all major society guidelines (British Society of Gastroenterologists (BSG), European Crohn’s and Colitis Organization (ECCO), European Society of Gastrointestinal Endoscopy (ESGE), American Gastroenterological Association (AGA)) [29–32].

European guidelines (ESGE, ECCO and BSG) recommend the routine use of pancolonic chromoendoscopy with targeted biopsies for neoplasia surveillance in patients with long-standing colitis [29–32]. However, chromoendoscopy technique is unappealing as it prolongs the procedure time in addition to being cumbersome as well as requiring sufficient expertise [33].

EC has the advantage over traditional chromoendoscopy as the image enhancement mode can be activated by push-button technology, and it is cheaper, quicker and more user-friendly.

Two randomized controlled trials compared high-definition (HD) NBI to CE. In a tandem study performed by Pellise et al., patients with long-standing colitis (*n* = 60) were randomized with HD NBI or CE [34]. The study showed no significant difference in dysplasia detection, and although NBI showed a numerically higher miss rate for dysplastic lesions when compared to CE (13.6 vs 31.8 %), this was not statistically significant [34]. A multicentre parallel randomized study involving 108 patients with chronic colitis showed a comparable detection rate for dysplastic lesions (18 with NBI and 26 with CE, *p* = 0.385) [35]. Efthymiou et al. performed a back to back study with HD NBI followed by CE in patients with long-standing colitis (>8 years duration) [36]. Overall, CE detected more lesions than NBI (131 vs 103, *p* < 0.001); however, most of them were non-dysplastic. There was no difference in dysplasia detection—CE detected 23 dysplastic lesions in 11 patients and NBI 20 lesions in 10 patients (*p* = 0.18). Similarly, studies comparing NBI with standard WLE failed to show any improvement in the detection of dysplasia [37, 38].

To date, NBI has failed to show any improvement in the detection of dysplasia in IBD when compared to WLE or CE. There are no published data available using FICE, i-Scan and BLI in colitis surveillance.

In summary, the current evidence does not support the use of EC in surveillance in IBD at present.

### Polyp characterization and optical diagnosis

The current practice mandates that all small polyps detected during colonoscopy should be resected and sent for histopathological assessment to determine the histopathological type ( adenoma, hyperplastic or more recently serrated lesions), as colonoscopic surveillance intervals are influenced by the number, size and histological features of polyps (i.e. adenoma, presence of villous features and high-grade dysplasia) [39, 40]. The majority of polyps detected at colonoscopy are diminutive (<5 mm) and rarely harbour cancer or high-risk features such as high-grade dysplasia or villous component [41, 42, 43••].

As diminutive polyps are of such limited significance, being able to diagnose adenomas in vivo would allow for them to be resected and discarded, saving the costs associated with histopathology. Furthermore, diagnosing distal hyperplastic polyps in vivo would allow for these to be left in situ (diagnose and disregard) reducing the risks associated with polypectomy [43••]. Estimates suggest that by implementing resect and discard policy savings of $33 million to $1 billion could be made annually in the USA alone [44]. Recent generation of high-definition instruments with virtual or electronic chromoendoscopy has enabled endoscopists to attempt to predict the histological type of polyps in vivo.

### NBI

**Fig. 1:**
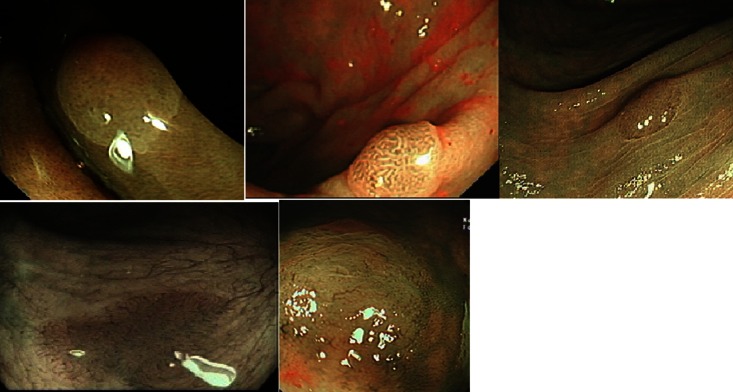
NBI images of polyps. Clockwise: NBI images of hyperplastic polyp, adenoma, adenoma, SSA/P and SSA/P.

Majority of the studies assessed NBI, a blue light optical imaging modality which enhances the mucosal architecture, especially vasculature. Vascular density and pattern differ between neoplastic (adenoma) and non-neoplastic (hyperplastic) polyps, allowing an endoscopist to use NBI to differentiate between the two (Fig. 1) [45]. A group of international experts have developed and validated a system to characterize small colonic polyps: the NBI International Colorectal Endoscopic (NICE) Classification (Table 2) [46]. This classification uses mucosal colour, vessel and surface pattern to differentiate between hyperplastic polyps and adenoma (Table 2). It is applicable for non-magnified NBI imaging. Authors made a diagnostic prediction with high confidence in 75 % of the 236 polyps (82 % in adenomas and 64 % in hyperplastic). These diagnoses achieved accuracy of 88 %, sensitivity of 98 % and negative predictive value of 95 % for diminutive polyps [46]. A meta-analysis by Wanders and colleagues evaluated real-time optical diagnosis by NBI and showed an overall sensitivity 91 % (95 % CI 88.6–93.0), specificity 85.6 % (81.3–89.0) and real-time negative predictive value 82.5 % (75.4–87.9) [47]. However, most of the studies in the meta-analysis were carried out by expert endoscopists in academic centres [47]. Ladabaum and colleagues prospectively evaluated real-time optical diagnosis of polyps with NBI by community-based gastroenterologists [48]. This study showed inferior results when compared to the academic centres with sensitivity, specificity and negative predictive values of 85, 78 and 91 %, respectively. New NBI system with near focus might offer a solution for this discrepancy as near-focus image may provide a more detailed view of the surface architecture. Singh et al. assessed 149 polyps using Olympus 190 series Exera III NBI system with dual focus (DF) capabilities [49]. The overall accuracy of NBI-DF when compared to final histopathology was 97 %. In addition, post-polypectomy surveillance interval when based on optical diagnosis was accurate in 97 % of cases with high negative predictive value (100 %) for diminutive rectosigmoid polyps.

**Table 2:** NICE classification

Colour	Same or lighter than background	Browner relative to background
Vessels	None or isolated lacy vessels coursing across the lesion	
Surface pattern	Dark or white spots of uniform size or homogenous absence of pattern	Oval, tubular or branched white structures surrounded by brown vessels
Most likely pathology	Hyperplastic	Adenoma

Adapted from Hewett DG, Kaltenbac T, Sano Y, et al. Validation of a simple system for endoscopic diagnosis of small colorectal polyps using NBI. Gastroenterology 2012;143:599–607.e1; with permission

### SSA/P

**Fig. 2:**
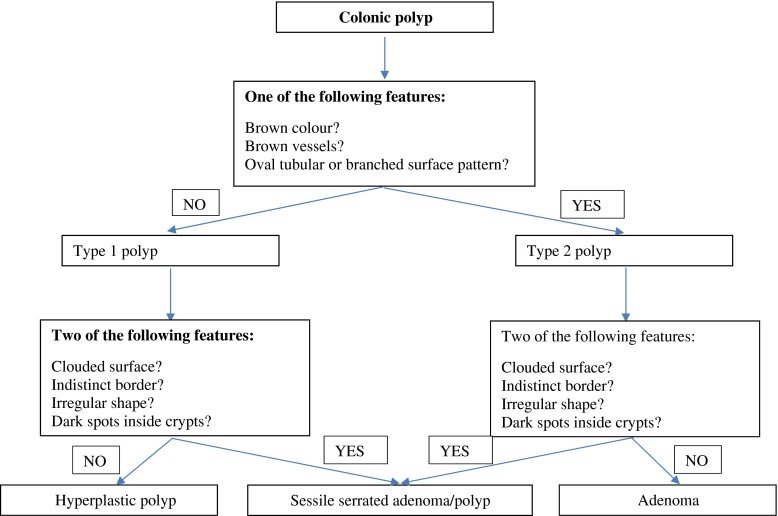
WASP classification. Adapted from IJspeert JE, Bastiaansen BA, van Leerdam ME, et al. Development and validation of the WASP classification system for optical diagnosis of adenomas, hyperplastic polyps and sessile serrated adenomas/polyps. Gut 2015;0:1–8; with permission.

It is often difficult if not impossible to differentiate hyperplastic polyps (HP) from SSA/P on white light endoscopic examination. Yamada et al. performed a retrospective image evaluation study in which 242 NBI lesions were evaluated by 2 experienced endoscopists [50••]. Multivariate analysis demonstrated that the presence of Dilated and ranching vessels (DBV) on the surface had a 2.3-fold odds ratio (95 % CI 0.96–5.69) among SSA/P compared to HPs. When DBV, proximal location and polyp size >10 mm were combined, the positive predictive value reached 92 % for SSA/P making it promising for the optical diagnosis of serrated lesions. Recently, IJspeert and colleagues developed a new classification system for in vivo differentiation of adenomas, HPs and SSA/P; the Workgroup serrAted polypS and Polyposis (WASP) classification (Fig. 2) [51••].

### FICE

Teixeira and colleagues proposed an endoscopic classification of the superficial capillary-vessel pattern of colorectal lesions to differentiate neoplastic polyps form non-neoplastic polyps: types 1 and 2 are hyperplastic polyps, types 3 and 4 are adenomas and type 5 capillary pattern indicates invasive cancer [52]. The authors analysed 309 colorectal lesions using the Teixeira capillary-vessel pattern classification and found 99.2 % sensitivity and 94.9 % specificity for neoplastic lesions [52].

Large prospective UK series by Longcroft-Wheaton et al. compared FICE with WLE and found that FICE improved the accuracy of in vivo diagnosis of adenoma from 75 to 88 % (*p* < 0.001) [53]. When using FICE, endoscopists predicted post-polypectomy surveillance interval with accuracy of 97 %. The previously mentioned meta-analysis by Wanders et al. examined 14 studies which used FICE to distinguish neoplastic polyps from non-neoplastic polyps in vivo during screening colonoscopies [47]. FICE showed a sensitivity of 91.8 % and negative predictive value of 83.7 %.

### i-Scan

A recent meta-analysis of 11 studies using i-Scan to differentiate neoplastic polyps from non-neoplastic polyps, which included 925 patients with 2312 polyps, demonstrated a sensitivity of 91.5 % and specificity of 92.1 % [54].

### BLI

**Fig. 3:**
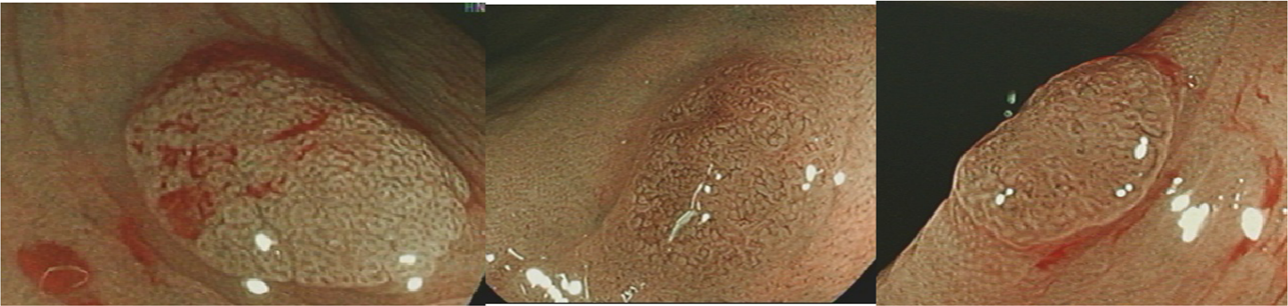
BLI images of polyps. Clockwise: BLI images of hyperplastic polyp, adenoma and adenoma.

Blue laser imaging (BLI) by Fujinon LASEREO system allows for narrow band observation. The BLI light is useful for acquiring mucosal surface information such as vascular and surface patterns (Fig. 3). Yoshida et al. retrospectively examined 314 colorectal polyp images [55]. The overall diagnostic accuracy of BLI without magnification for differentiating between neoplastic and non-neoplastic polyps (<10 mm) was 95.2 %, which was greater than that of WLE (83.2 %).

In summary, EC is a promising imaging technology to make an accurate optical diagnosis in vivo. NBI has been extensively evaluated and is highly accurate in expert hands in predicting the histopathology of small polyps. It is a user-friendly and easy to apply technique with a push of a button. FICE and i-Scan showed encouraging results. BLI technique needs to be explored further in randomized controlled trials.

### Implementation of optical diagnosis

The initial clinical studies suggested that optical diagnosis for diminutive colorectal polyps could be feasible and safe in routine clinical practice. However, recent reports suggest that optical diagnosis performed by non-academic gastroenterologists could not reproduce similar results. Therefore, certain key steps should be followed before translating optical diagnosis into the community practice.

Consequently, the American Society of Gastrointestinal Endoscopy (ASGE) developed a Preservation and Incorporation of Valuable Endoscopic Innovations (PIVI) statement for in vivo endoscopic assessment of diminutive polyps [56•]. Furthermore, a recent update on ‘implementation of optical diagnosis of colorectal polyps’ raised the issues of training, competency assessment and documentation before optical diagnosis could be implemented into routine clinical practice [57].

### Training and competency assessment

The knowledge and skills required to perform optical diagnosis can be learned by people with varying experiences in colonoscopy. Ignjatovic et al. from St Mark’s Hospital used a still image-based teaching module to differentiate between adenomas and hyperplastic polyps [58]. They showed improved accuracy and specificity of optical diagnosis for all group of participants which included novices, trainees and experienced colonoscopists after a short training module (*k* = 0.56, 0.70 and 0.54, respectively). Recently, WASP classification showed some promising results in differentiating HPs from SSA/P [51••]. In a still image evaluation setting, the introduction of the WASP classification significantly improved the accuracy of optical diagnosis overall and proved to be sustainable after 6 months. Overall, the accuracy of optical diagnosis improved from 63 % before training to 79 % after training (*p* < 0.001) and remained at 76 % after 6 months. This study highlighted the importance of continued training and re-assessment of individuals who perform optical diagnosis to maintain the threshold. The fact ex vivo training does not necessarily translate into actual clinical practice was highlighted by the study by Ladabaum and colleagues [48]. In an evaluation of real-time optical diagnosis with NBI, only 25 % of gastroenterologists assessed polyps with ≥90 % accuracy while more than 83 % of gastroenterologists performed with >90 % accuracy ex vivo.

These findings suggest that in vivo training with feedback and assessment is needed before non-academic gastroenterologists are certified to perform optical diagnosis in routine clinical practice.

### Documentation

High-quality image of the polyp assessed by optical modality should be taken for quality assurance and medicolegal purposes. The question of whether high-quality video clips are necessary for documentation remains to be answered.

## Conclusion

The routine use of EC is becoming more popular among the western endoscopists as an alternative to traditional chromoendoscopy due to its wider availability and user-friendly nature. Although there is uncertainty whether NBI improves overall adenoma detection, there are trends that suggest that it may improve the detection of diminutive polyps and sessile serrated polyps. Furthermore, NBI is superior in characterizing polyps especially sessile serrated adenomas when compared to white light. Recent studies showed promising results for i-Scan as well. There is an unprecedented focus on real-time prediction of histology using EC. This ability would allow the endoscopists to predict the surveillance intervals immediately after the colonoscopic examination and could potentially lead to significant cost savings to the health care system. However, the implementation of optical diagnosis in clinical practice needs to be agreed by the relevant gastroenterological societies. Robust and rigorous training, accreditation and quality assurance aspects need to be agreed and validated.
